# Is adding resources always beneficial? Multiplicative impact of psychological capital and goal-oriented climate on Spanish public worker satisfaction and engagement

**DOI:** 10.3389/fpsyg.2024.1418409

**Published:** 2024-07-18

**Authors:** Pedro Antonio Díaz-Fúnez, Giuseppina María Cardella, Brizeida Raquel Hernández-Sánchez, José Carlos Sánchez-García, Miguel Ángel Mañas-Rodríguez

**Affiliations:** ^1^IPTORA Research Team, Department of Psychology, University of Almeria, Almería, Spain; ^2^AFIDE, Department of Psychology, University of Salamanca, Salamanca, Spain

**Keywords:** resources, motivational process, psychological capital, public worker satisfaction, goal-oriented climate, employee engagement, job demands resources model

## Abstract

**Introduction:**

The motivation process from personal resources to commitment of administrative employees is still little studied. This article uses the Job Demands-Resources model to investigate how the multiplicative effect of personal resources and a goal-oriented climate among public employees influences their satisfaction and engagement at work. Specifically, it proposes a model where the influence of psychological capital on engagement is mediated by job satisfaction and moderated by the goal orientation climate.

**Method:**

A total of 326 employees of the administrative staff of a Spanish Public Administration answered a self-reported survey. Partial Least Square-Structural Equation Modeling (PLS-SEM) approach was used to evaluate the validity and reliability of the data, as well as, to test the hypotheses formulated.

**Results:**

The SEM results show the positive impact of psychological capital on employee engagement, and the mediating role of job satisfaction in this relationship. Furthermore, the existence of a goal-oriented climate negatively moderated the relationship between Psychological Capital and Job Satisfaction, reducing the mediation effect.

**Discussion:**

These findings open new doors for future research in the necessary adaptation of human resource policies to improve the motivation process in the public administration context.

## 1 Introduction

Changes within the public sector have remained continuous over the past three decades (Pecino et al., 2019). These alterations are particularly noticeable during moments of societal-wide impact, such as economic downturns, emergencies, or extraordinary occurrences, such as the COVID-19 pandemic (Del Carmen Giménez-Espert et al., 2020; Jasiński, 2020). Within this framework, there is a growing demand for public workers to extend beyond their traditional roles. One approach to address this demand has involved a focus on leveraging personal resources and fostering positive emotions among these workers (Luu, 2020).

In Spain, the public sector is characterized by numerous demands (Triguero-Sánchez et al., 2021). Alongside the extensive bureaucracy and limited flexibility in the enforcement of regulations (Burzaco-Samper et al., 2013), there exists a wide array of regulations applicable depending on the type of entity (Arenilla-Sáez, 2017). These elements distinguish the context of public administrations in Spain, particularly in their satisfaction process.

Employee Engagement is a positive emotional and motivational state experienced in the workplace, characterized by vigor, absorption, and dedication (Rothbard, 2001; Schaufeli et al., 2002). Despite numerous studies exploring its causes and the beneficial outcomes it yields for organizations, research within the public sector remains scarce (Vigoda-Gadot et al., 2013; Zahari and Kaliannan, 2022). Consequently, there is a notable gap in understanding work engagement specifically within public sector settings. Consequently, it is imperative to investigate how enhancing it can impact worker engagement (Costantini et al., 2017).

Bakker and Demerouti (2008) propose that psychological capital is one of the determinants of the motivational process. This concept integrates elements of positive psychology and occupational psychology (Youssef and Luthans, 2007; Luthans and Avolio, 2009; Avey et al., 2010). This proposition has led researchers to explore this variable as one of the main predictors of employee engagement in public sector entities (George et al., 2021).

However, the impact of Psychological Capital on work engagement might be influenced by other psychological factors linked to the work environment, such as perceptions and emotions. This opens an intriguing line of inquiry to determine whether the direct positive effects of job resources maintain a multiplicative effect on the motivational process when they coexist in the context (Bakker et al., 2023). Numerous studies have indicated that work engagement stems from the positive psychological resources of employees (Demerouti and Bakker, 2011; Yeh, 2013), which are tied to job satisfaction and the organizational climate. Organizational climate, characterized by shared perceptions of workplace norms, priorities, and expectations among employees within a collective (Schneider et al., 2013), has shown significant correlations with individual and group outcomes across various organizational contexts (for example, Christian et al., 2009; Whitman et al., 2010; Hong et al., 2013), showing a great relevance, in policies aimed at enhancing goal orientation in public administration (Vandewalle et al., 2001).

This study aims to investigate how the multiplicative effect of personal resources and a goal-oriented climate among public employees influences their satisfaction and engagement at work. Specifically, it proposes a model where the influence of psychological capital on engagement is mediated by job satisfaction and moderated by the goal orientation climate.

Public sector workers may have missions that offer opportunities to fulfill altruistic or higher-order needs, potentially boosting workforce motivation (Perry and Hondeghem, 2008). However, the bureaucratic nature and conflicts within public organizations could impede the realization of these opportunities (Nezhina et al., 2021). Additionally, the lack of specific organizational goals, which is common in public sector organizations, may negatively affect psychological responses (Jin et al., 2016; Kjeldsen and Hansen, 2016).

The Job Demands-Resources (JD-R) theory lays the groundwork for understanding how work influences the psychological responses of employees (Bakker and Demerouti, 2014). According to this theory, all jobs, whether in public or private organizations, involve both demands and resources. High job demands can negatively impact psychological well-being by causing mental stress, emotional strain, and physical fatigue. These demands may become stressors when they exceed an individual’s capacity to meet them, requiring significant effort to maintain performance levels. On the other hand, job resources help alleviate job demands and their associated physiological and psychological costs. They contribute to achieving work goals and fostering personal growth, learning, and development (Demerouti et al., 2001).

Furthermore, empirical studies are necessary to understand whether employee work engagement is primarily influenced by the interplay between various demands and resources (Cooke et al., 2018). Therefore, it is important to identify the factors that precede work engagement, as it can vary depending on the specific job, organization, or group context (Saks, 2006).

A significant amount of research has highlighted the importance of resources in enhancing employees’ work engagement. These findings suggest that resources play a central role in promoting work engagement. In fact, resources have been identified as the most influential factors positively affecting work engagement, as they enable employees to initiate a process of personal growth and learning (Bailey et al., 2015). According to the motivational process outlined in the JD-R theory, resources serve a dual purpose. They are inherently motivating by satisfying employees’ basic human needs such as autonomy, belongingness, and competence, thereby promoting their knowledge and mastery. Additionally, they have extrinsic motivational potential by providing instrumental support that helps employees achieve their work goals (Bakker and Demerouti, 2017). However, empirical findings regarding the strength of the relationship between engagement and different types of resources vary.

One of the paramount assets within the realm of Work and Organizational Psychology is Psychological Capital (PsyCap). Initially conceptualized by Luthans and colleagues (Luthans, 2002; Luthans et al., 2007a,b), this construct delineates the psychological disposition of an individual characterized by several facets: possessing the confidence (efficacy) requisite for undertaking and exerting effort in overcoming challenging tasks; harboring a positive outlook (optimism) regarding both present and future success; persisting toward objectives and, when necessary, adapting strategies (hope) to facilitate achievement; and, when confronted with obstacles and adversity, demonstrating resilience by enduring and rebounding to attain success (Luthans et al., 2007b).

PsyCap emerges as a pivotal determinant of work engagement (Rothmann, 2008; Simons and Buitendach, 2013). Optimism, characterized by individuals’ ability to perceive favorable aspects of current and future events and correlate them with performance outcomes, plays a significant role. Literature suggests that cynical individuals tend to exhibit lower levels of optimism; however, optimism can mitigate the effects of cynicism, fostering greater dedication and attenuating the adverse effects of various stressors (Bakker and Schaufeli, 2008). The presence of optimistic outlooks regarding positive outcomes facilitates psychological openness, enabling individuals to immerse themselves in their surroundings and consequently fostering heightened engagement (Kahn, 1990). Overall, optimism correlates more strongly with engagement facets such as dedication and absorption.

Psychological resources serve as a reservoir that furnishes elements such as resilience, essential for motivation and work engagement, indicative of an individual’s vigor or robustness (Bakker and Leiter, 2010). Within the scholarly discourse, resilience is recognized as a supplementary asset capable of mitigating the excessive negative impact of job demands and burnout, functioning as a backup or additional resource (Troy and Mauss, 2011). Resilience, characterized as a state with the capacity to influence not only the present moment but also to mitigate past stressors, is a crucial factor in navigating challenges effectively (Hetzel-Riggin et al., 2019). The relationship between resilience and work engagement is perceived as proportional, wherein heightened resilience enables individuals to effectively manage job demands, stressors, and maintain overall control. It is justifiable to assert that resilience is intricately linked to the characteristics of job engagement. Building upon this rationale, it is reasonable to hypothesize that individuals who effectively leverage their PsyCap will demonstrate enhanced performance, thereby fostering better work engagement. Consequently, the following hypotheses are formulated to empirically examine the effect of PsyCap on work engagement.

*H1*: There is a positive effect of PsyCap on Employee Engagement.

Within the organizational framework, personal resources intersect with various other resources and emotional responses to shape an employee’s emotional attachment to their organization. One such example is job satisfaction, which refers to a pleasurable emotional state stemming from the appraisal of one’s job as fulfilling or facilitating the achievement of valued outcomes (Locke, 1969). According to social exchange theory, individuals who experience high levels of job satisfaction are inclined to reciprocate by increasing their engagement with work as a form of reward to the organization (Kieserling, 2018).

Empirical evidence supports the notion of a significant correlation between job satisfaction and work engagement. Yalabik et al. (2017) found that satisfaction with one’s current work situation is a pivotal driver of all dimensions of work engagement, indicating a robust relationship between job satisfaction and engagement. Similarly, a study by Zhang and Li (2020) involving 212 corporate employees demonstrated a significant positive correlation between job satisfaction and work engagement.

Several findings underscore the importance of fostering job satisfaction as it contributes positively to work engagement, thereby enhancing organizational performance and employee well-being (Bakker and Demerouti, 2008; Wefald and Downey, 2009; Alarcon and Edwards, 2011; Yeh, 2013).

*H2*: There is a positive effect of Job Satisfaction on Employee Engagement.

Among the myriad individual and organizational factors influencing job satisfaction, psychological capital emerges as one of the most significant individual factors (Kong et al., 2018). Positive psychological resources, encapsulated within PsyCap, play a pivotal role in enabling employees to effectively navigate their demanding work routines (Amunkete and Rothmann, 2015). Job satisfaction is posited to potentially mediate the impact of both job-related (Azanza et al., 2013) and psychological resources (Wefald and Downey, 2009; Badran and Youssef-Morgan, 2015) on various positive outcomes for employees.

While existing research has established a link between job satisfaction and employee engagement (Bakker and Demerouti, 2008; Wefald and Downey, 2009; Alarcon and Edwards, 2011; Yeh, 2013), it remains unclear whether the mediating role of job satisfaction consistently persists when accounting for personal resources in predicting work engagement.

*H3*: There is a positive effect of PsyCap on Job Satisfaction.

*H4*: Job Satisfaction has a mediated role between PsyCap and Employee Engagement.

Indeed, not all resources necessarily yield a positive impact on employees’ emotional responses within the organizational context. Various theoretical models shed light on the predictors and processes influencing public employees’ experiences and their association with negative emotional bonds with the organization. For instance, Self-Determination Theory, as expounded by Ryan and Deci (2017), posits that work environments can fulfill employees’ basic needs by fostering their growth, well-being, and optimizing achievement outcomes. However, when these needs are not met, it can have a detrimental effect on employees’ emotional responses. Similarly, goal orientation theory offers an additional framework for understanding how individuals’ orientations toward achievement influence their commitment. This theory delineates diverse motivational responses among employees based on their individual preferences in achievement-oriented situations (Vandewalle et al., 2001). These theoretical perspectives highlight the nuanced interplay between organizational environments, employee needs, and motivational orientations, elucidating the complexity of emotional bonds within the workplace.

The achievement-oriented climate represents a resource that, paradoxically, may exert a negative effect on employee well-being. Vandewalle and Cummings (1997) delineated three styles of achievement orientation: Learning Goal Orientation (LGO), Performance-Approach Goal Orientation, and Performance-Avoidance Goal Orientation. LGO involves a focus on enhancing one’s competence through acquiring new skills, effectively managing novel situations, and learning from experience. Performance-Approach Goal Orientation centers on optimizing one’s competence and attaining positive outcomes. However, if employees fail to discern the positive effects of these goals, they may gravitate toward Performance-Avoidance Goal Orientation, perceiving it as detrimental to the maintenance of their competencies. Meta-analytic research supports this classification of goal orientations and underscores its stability over time (Payne et al., 2007). Depending on the specific achievement orientation adopted by public employees, it may elicit varying effects on their emotional states.

*H5*: Goal-oriented Climate moderates the relationship between PsyCap and Job Satisfaction, such that the relationship between PsyCap and Job Satisfaction is weaker in a goal-oriented climate.

**Figure 1:**
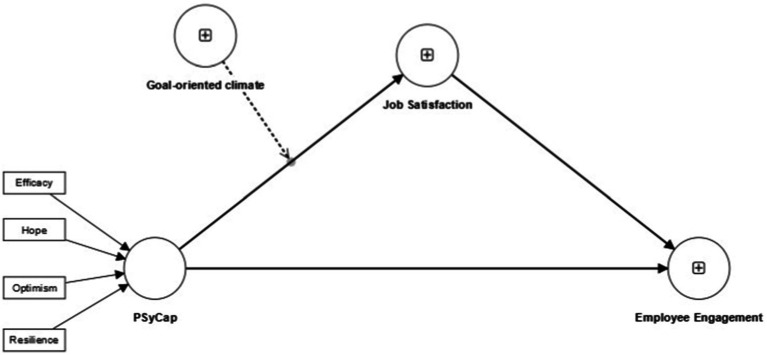
Conceptual model.

Based on the employee’s level of Psychological Capital (PsyCap), this study investigates the influence of PsyCap as a personal resource for public sector employees on their engagement development. It posits that this influence is moderated by the organizational climate and the resultant level of job satisfaction achieved by the employee, contingent upon their PsyCap (Figure 1).

## 2 Materials and methods

### 2.1 Sample

The sampling technique was carried out based on a non-probability sampling method with purposive sampling technique. Although this type of sampling can represent a problem in terms of generalizability of the results (for example, generalization is only possible to the population defined by the sample selection criteria, in our case Spanish public employees), it appears more effective when it is necessary to study a certain cultural context with certain characteristics (Andrade, 2021).

The sample was composed of 326 employees of the administrative staff of a Public Administration in Salamanca (a Spanish city in the Autonomous Community of Castilla y León), of which 204 were females (62.6%) and 122 were males (37.4%). Participants’ ages were included with ranges from under 30 to over 60 years old at the time of sending the questionnaire. Specifically, most of the sample (56.8%) was between 51 and 60 years old, and more than half (54%) possessed a degree certificate qualification.

Finally, the legal status of the participants was 92.9% that of career civil servant, with a length of service, in the majority of cases, more than 26 years (35%), and especially operating in the public service delivery sector (53.8%).

Table 1 offers the sociodemographic characteristics of the population under study.

**Table 1:** Demographic characteristics of respondents.

Items	Category	Frequency (%)
Gender	Male	122 (37.4)
	Female	204 (62.6)
	Under 30	9 (2.7)
Age	31–40 years	33 (10.2)
	41–50 years	63 (19.4)
	51–60 years	185 (56.8)
	Above 60 years	36 (11)
Education level	Elementary school	6 (1.8)
	Middle school	8 (2.5)
	High certificate	57 (17.5)
	Degree certificate	176 (54)
	Postgraduate certificate	79 (24.2)
Legal status	Career civil servants	302 (92.9)
	Temporary civil servants	16 (4.7)
	Permanent employees	7 (2.1)
	Temporary workers	1 (0.3)
Length of service	1–4 years	48 (14.7)
	5–10 years	36 (11)
	11–20 years	77 (23.6)
	21–25 years	51 (15.6)
	Above 25 years	114 (35)
Service Types (Roles)	HR Management	38 (11.7)
	Administrative and Financial Management	92 (28.1)
	Public Service Delivery	175 (53.8)
	Other Areas/Services	21 (6.4)

#### 2.1.1 Ethics statement

The participation was voluntary and anonymous, which included handling a letter of informed consent, explaining the objectives of the study, determining the risks and benefits for the study participants, without limiting their freedom (Kang and Hwang, 2023). All participants gave their informed consent for inclusion before they participated in the study. Participants completed the questionnaire in approximately 20 min and did not receive any credit or financial compensation for participating in the study. The study was conducted in accordance with the Declaration of Helsinki. Ethical review and approval were not required for the study on human participants in accordance with local legislation and institutional requirements.

### 2.2 Measures

The survey was conducted online using a Google Form, from January to March 2023. Online questionnaires present some advantages, they are useful for collecting data on behaviors, experiences, attitudes, beliefs and values (Dawson, 2020) and, moreover, they make the administration process more agile as they do not require the presence of the interviewers (Duffy et al., 2005). The questionnaires used in this study are detailed below.

#### 2.2.1 Dependent variable

Employee Engagement has been measured using a short version of the Utrecht Work Engagement Scale developed by Schaufeli et al. (2002), and adapted in Spanish version by Pujol-Cols and Arraigada (2018). The UWES is a 7-item instrument, again Likert-type, ranging from 0 (completely disagree) to 7 (completely agree). Theoretically, 3 items measure vigor (greater energy level with enough mental resilience), 2 others are indicators of dedication (inspiration and enthusiasm about one’s work), and 2 conform to absorption (well-connected and engrossed with work throughout).

Example items of vigor are: “At my work, I feel bursting with energy.” Dedication includes items such as “I find the work that I do full of meaning and purpose.” Finally, the dimension of absorption is tapped by items such as “Time flies when I’m working.” In our study, and in line with past research (Fong and Ng, 2011; Panthee et al., 2014), we considered scale as a single factor as it fit better in our model (see exploratory factorial analysis in the results section).

#### 2.2.2 Independent variable

Psychological capital was measured with the Spanish version of the Psychological Capital Scale by Luthans et al. (2007a). The PCQ12 questionnaire was adapted by León- Pérez et al. (2017). The scale is composed of 12 items, with a Likert-type response format, from 1 (disagree) to 6 (agree) and four sub-dimensions: Efficacy (confidence in one’s self and exerting efforts to succeed, 3 items) hope (perseverance to achieve the goal and align when necessary, 4 items), resilience (tendency to bounce back from adversity, 2 items), and optimism (looking at the bright side through positive attributes, 2 items).

Example of the items are: “I feel confident analyzing a long-term problem to find a solution,” “I usually manage difficulties one way or another at work,” “When things are uncertain for me at work, I usually expect the best,” and “There are lots of ways around any problem.”

#### 2.2.3 Mediating variable

Job satisfaction was measured using the Job Satisfaction Scale developed by Warr et al. (1979), using the version adapted to Spanish by Baluarte (2014). The scale measures both the intrinsic and the extrinsic nature of employees’ satisfaction with their job. The respondents were asked to 10 items with a seven-point Likert scale from 1 (very dissatisfied) to 7 (very satisfied). Example of items are: “I feel fairly well satisfied with my present job,” and “I am satisfied with my job for the time being.”

#### 2.2.4 Moderating variable

The goal-oriented climate assessment was carried out using the FOCUS-93 (First Organizational Climate/Culture Unified Search) questionnaire (Van Muijen et al., 1992) in its short and Spanish version (González-Romá et al., 1996). This instrument measures climate across 4 dimensions (support, rules, innovation and goals). For the purposes of this study, the goals subscale was used. Goal-oriented climate assessment scale was measured using 3 Likert items whose scale ranges from 1 (totally disagree) to 7 (totally agree). Examples of items are: “How often does management specify the targets to be attained?,” “Is it clear how performance will be evaluated?”

Table 2 presents the descriptive statistics and the correlation among the variables in the study.

**Table 2:** Descriptive statistics and correlation matrix.

	Mean	Std. Dev.	Median	1	2	3	4
1. PsyCap	4.42	0.821	4.50	-			
2. Job Satisfaction	5.01	1.241	5.30	0.497**	-		
3. Goal-oriented Climate	4.12	1.627	4.33	0.383**	0.689**	-	
4. Empl. Engag.	4.84	1.518	5.14	0.548**	0.808**	0.605**	-

**p < 0.01; *p < 0.05.

### 2.3 Statistical analysis

All analyses were performed using the SmartPLS 4 software with bootstrap method (5,000 re-samples) (Ringle et al., 2024) and IBM SPSS 29.0 statistical software packages (IBM Corp, 2023). The Kaiser-Meyer-Olkin Measure of Sampling Adequacy and Bartlett’s Test of Sphericity were used to assess the suitability of the data for factor analysis. Exploratory factor analysis (EFA) was performed using principal components extraction and eigenvalues > 1, with varimax rotation in order to test the theoretical structure of the questionnaires.

Additionally, according to Hair et al. (2019), two-stage analytical procedures were recommended to analyze the data. In the first stage of confirmatory composite analysis (CCA), we assessed the measurement model by evaluating the estimates of internal consistency reliability and convergent and discriminant validity. In the second stage the partial least squares (PLS) approach was used to evaluate the validity and reliability of the data, as well as, to test the hypotheses formulated.

Partial Least Square-Structural Equation Modeling (PLS-SEM) is the preferable approach when researchers focus on prediction and theory development. Secondly, PLS-SEM, unlike CBM-SEM, is robust to non-normality of data, and therefore is methodologically the preferable tool when working with Likert-type scales (Sarstedt et al., 2011; Hair et al., 2019).

However, an analysis of the normality of the data was still carried out for greater transparency and robustness of the results. Since the sample size >300, empirical normality tests such as Kolmogorov–Smirnov (KS) and Shapiro Wilk (SW) cannot be utilized since they are insufficient for samples larger than 300 (Mishra et al., 2019). Kim (2013) emphasized the importance of skewness and kurtosis testing for a valid normality test, regardless of the sample size. For sample sizes bigger than 300, however, rely on histograms and absolute values of skewness and kurtosis instead of z-values. Additionally, when employing SEM, appropriate skewness values are between ±3, whereas acceptable kurtosis values are between ±10 (Brown, 2006). Hair et al. (2010) defined normal data as having skewness between ± and kurtosis between ±7. For Mishra et al. (2019), an absolute skewness value ≤2 or an absolute kurtosis ≤4 may be used as reference values to determine a considerable normality.

The results showed that the data were approximately normally distributed as the skewness and kurtosis of all variables were within ±2 and ± 4, respectively. Specifically: CapPsy: skewness −0.400 (std. err. 0.135), kurtosis −0.018 (std. err. 0.269); Goal-orientated Climate: skewness −0.151 (std. err. 0.135), kurtosis −0.990 (std. err. 0.269); Job Satisfaction: skewness −0.701 (std. err. 0.135), kurtosis −0.059 (std. err. 0.269); Engagement: skewness −0.640 (std. err. 0.135), kurtosis −0.225 (std. err. 0.269).

## 3 Results

### 3.1 Exploratory factor analysis

Before analyzing the measurement model, we decided to perform an exploratory factor analysis (EFA) to ensure its validity and reliability. In exploratory factor analysis with a varimax rotation, we observed that the four dimensions of psychological capital factor accumulate 74% of the variance (KMO = 0,863; *p* < 0.001; Eigenvalue = 1.180). Table 3 shows the factor loadings. As the table shows, all loadings were relatively high, ranging from 0.66 to 0.91.

**Table 3:** Exploratory factor analysis: factor loadings of four-dimensional structure of PsyCap.

	Component			
Items	Efficacy	Hope	Resilience	Optimism
P1.1	0.814			
P1.2	0.862			
P1.3	0.788			
P1.4		0.665		
P1.5		0.785		
P1.6		0.680		
P1.7		0.914		
P1.8			0.901	
P1.9			0.860	
P1.10			0.766	
P1.11				0.743
P1.12				0.828

Extraction method: principal component analysis. Rotation method: varimax.

All other variables were confirmed as single-factor (*Job Satisfaction:* KMO = 0,926; *p* < 0.001; Eigenvalue = 5.746; Fraction of variance in % = 57.463; factor loadings ≥0.54); (*Goal-oriented Climate*: KMO = 0,736; *p* < 0.001; Eigenvalue = 2.462; Fraction of variance in % = 82.067; factor loadings ≥0.88; *Engagement:* KMO = 0,898; *p* < 0.001; Eigenvalue = 5.660; Fraction of variance in % = 80.858; factor loadings ≥0.83). In addition, the overall model showed good model fit (SRMR = 0.053, NFI = 0.941) indicating construct validity.

### 3.2 Measurement model: confirmatory composite analysis and discriminant analysis

Since our model contains both reflective and formative constructs, we had to treat them differently. To assess the convergent validity of the reflective constructs (PsyCap sub-dimensions, Goals, Job Satisfaction, and Employee Engagement), a PLS algorithm with factor weighting scheme and 300 iterations was performed to generate the values of factor loadings, composite reliability (CR), Dijkstra-Henseler reliability (Rho_a), and average variance extract (AVE).

According to Hair et al. (2019), the factor loading of each items on the respective construct should be greater than 0.704. Factor Loadings between 0.40 and 0.70 can be maintained if they do not negatively affect the validity and reliability of the model.

Composite reliability (CR), Cronbach’s alpha (CA) and coefficient Dijkstra-Henseler Rho_A are measures of internal consistency reliability and must exceed the threshold value of 0.70 (Dijkstra and Henseler, 2015). As shown in Table 3 the factor loadings of all observed variables gave satisfactory results. Furthermore, the reliability of CR, CA and Rho_a for latent variables exceeded the cutoff value of 0.70. Therefore, the reliability of all measurements was ensured at the item and construct level.

The assessment of construct validity was confirmed based on convergent validity and discriminant validity (Henseler, 2017). The measure of convergent validity is the AVE which measures that the variance of the construct can be explained by the indicators (Fornell and Larcker, 1981). The AVE must be greater than or equal to 0.50 and provides the amount of variance a construct obtains from its indicators in relation to the amount of variance due to measurement error; this means that each construct or variable explains at least 50% of the variance of the indicators (Fornell and Larcker, 1981). As demonstrated in Table 4, the AVE values for the constructs exceeded the recommended value of 0.50. Thus, convergent validity was ensured.

**Table 4:** Measurement model evaluation for reflective first-order constructs.

Variables	Indicators	Factor loadings	Cronbach’s alpha	Rho_a	Composite reliability	AVE
Efficacy	P1.1	0.872	0.828	0.829	0.897	0.745
	P1.2	0.876				
	P1.3	0.840				
Hope	P1.4	0.650	0.801	0.813	0.871	0.631
	P1.5	0.868				
	P1.6	0.825				
	P1.7	0.818				
Resilience	P1.8	0.677	0.797	0.722	0.833	0.627
	P1.9	0.802				
	P1.10	0.883				
Optimism	P1.11	0.894	0.731	0.732	0.732	0.788
	P1.12	0.881				
Job Satisfaction	P3.1	0.765	0.914	0.932	0.929	0.738
	P3.2	0.841				
	P3.3	0.882				
	P3.4	0.865				
	P3.5	0.797				
	P3.6	0.676				
	P3.7	0.655				
	P3.8	0.631				
	P3.9	0.690				
	P3.10	0.880				
Goal-oriented Climate	P2.1	0.918	0.891	0.894	0.932	0.821
	P2.2	0.920				
	P2.3	0.879				
Employee Engagement	P4.1	0.974	0.959	0.959	0.928	0.924
	P4.2	0.970				
	P4.3	0.934				
	P4.4	0.936	0.857	0.857	0.933	0.875
	P4.5	0.935				
	P4.6	0.930	0.834	0.835	0.923	0.857
	P4.7	0.922				

Discriminant validity was analyzed through the Fornell and Larcker (1981) criterion, according to which the square root of AVE in every latent variable should be more than other correlation values among the latent variables.

Table 5 revealed that the square root of AVE values is higher than the correlation values.

**Table 5:** Discriminant validity of the measurement model based on Fornell-Larcker criterion.

Construct	Efficacy	Hope	Resilience	Optimism	Job Sat.	Goal-oriented climate	Empl. Engag.
Efficacy	**0.863**						
Hope	0.357	**0.862**					
Resilience	0.410	0.408	**0.896**				
Optimism	0.323	0.719	0.567	**0.885**			
Job Satisf.	0.116	0.664	0.288	0.582	**0.859**		
Goal-oriented Climate	0.079	0.528	0.214	0.438	0.702	**0.906**	
Empl. Engag.	0.151	0.599	0.334	0.583	0.780	0.585	**0.906**

Diagonal values in bold are the square root of the variance shared between the constructs and their measures (AVE). Values below the diagonal elements are the correlations between constructs. Calculations not applicable to PsyCap.

More recently Henseler et al. (2015) proposed the Heterotrait-Monotrait ratio of correlations (HTMT) to analyze discriminant validity. The HTMT is defined as the mean value of the item correlations between the constructs versus the mean of the mean correlations for items measuring the same construct. An HTMT value greater than 0.90 (Gold et al., 2001) would suggest that discriminant validity is not present (Table 6).

**Table 6:** Discriminant validity of the measurement model based on Heterotrait-Monotrait ratio.

Construct	Efficacy	Hope	Resilience	Optimism	Job Sat.	Goal-oriented climate	Empl. Engag.
Efficacy	-						
Hope	0.455	-					
Resilience	0.511	0.515	-				
Optimism	0.436	0.713	0.801	-			
Job Satisf.	0.136	0.724	0.340	0.689	-		
Goal-oriented Climate	0.092	0.596	0.258	0.528	0.768	-	
Empl. Engag.	0.175	0.666	0.395	0.709	0.732	0.638	-

Calculations not applicable to PsyCap.

This study used the repeated indicator approach (Becker et al., 2012) to establish the reflective–formative higher-order construct of PsyCap. In the repeated indicators approach, all indicators of the lower-order components were assigned simultaneously to identify the higher-order component.

According to Hair et al. (2019) to establish the measurement model for the second-order formative construct two fundamental aspects are considered: (1) evaluation of collinearity problems, and (2) evaluation of the significance and outer weight of indicators.

The variance inflation factor (VIF) to evaluate the collinearity of the formative indicators was used. The VIF values for the 4 dimensions were less than 3 (Becker et al., 2015) indicating that collinearity was not an issue (Efficacy = 1.224, Hope = 1.782, Resilience = 1.551, and Optimism = 2.144).

Next, bootstrapping procedures with 5,000 re-sample were performed to assess the significance and relevance of the relationships between lower-order components and their higher-order components. The outer weight for the sub-dimensions was significant at *p* < 0.001. Specifically, Efficacy factor weights of 0.689 (*t* = 8.645), Hope factor weights of 0.638 (*t* = 8.347), Resilience factor weights of 0.541 (*t* = 6.232), and Optimism factor weights of 0.586 (*t* = 6,754). This reveals that the first-order construct significantly explains the second-order construct. Thus, the measurement model for the second-order formative was established.

### 3.3 Structural model and hypotheses testing: mediation and moderation

The results support our hypotheses. As can be seen in Table 7, PsyCap has a direct positive effect on engagement (H1: *β* = 0.213, *p* < 0.001) and Job Satisfaction (H3: *β* = 0.356, *p* < 0.001). The results also indicate that job satisfaction has a direct and stronger effect on employee engagement (H2: *β* = 0.682, *p* < 0.001). Furthermore, Job Satisfaction mediates the relationship between PsyCap and employee engagement (H4: *β* = 0.243, CI [0.169–0.318]). This result indicates that the effect of psychological capital on employee engagement is mediated by job satisfaction.

**Table 7:** Hypotheses testing.

Construct	*β*	*t*-Value	PCI	*f*^2^
Direct effect				
Employee engagement (*R*^2^ = 0.698)				
PsyCap → Employee engagement (H1)	0.213***	4.404	[0.115–0.304]	0.087
Job Satisfaction → Employee engagement (H2)	0.681***	16.229	[0.598–0.760]	0.891
Job satisfaction (*R*^2^ = 0.622)				
PsyCap → Job satisfaction (H3)	0.356***	6.948	[0.242–0.446]	0.228
Goal-oriented climate → Job satisfaction	0.499***	13.019	[0.425–0.573]	0.497
PsyCap*Goal-oriented climate → Job satisfaction (H5)	−0.106**	2.925	[−0.181–0.041]	0.037
Indirect Effect				
PsyCap → Job satisfaction → Employee engagement (H4)	0.243***	6.734	[0.169–0.311]	
Total effect (Direct and Indirect Effects)				
PsyCap → Employee engagement	0.455***	9.066	[0.340–0.541]	

PCI, Percentile Confidence Interval. Paths from hypotheses assessed by applying a one tailed test at 5% of significance level [5, 95%]. Bootstrapping based *n* = 5,000 bootstrap samples. ***p < 0.001, **p < 0.05.

Subsequently, we observed that a goal-oriented climate has a negative moderating effect on the relationship between PsyCap and Job Satisfaction (H5: *β* = −0.106, CI [−0.181- -0.041] see Table 7). Our results indicate that, within the Public Administration, a goal-oriented working climate negatively weakens the relationship between one’s psychological abilities and job satisfaction. Therefore, hypothesis 5 was also supported.

The results show that the total effect of the PsyCap on employee engagement is (*β* = 0.455, CI [0.340–0.541]) because it is the sum of the direct (*β* = 0.213, CI [0.115–0.304]) and indirect (*β* = 0.243, CI [0.169–0.311]) effects, where the indirect effect denotes the effect of the PsyCap on employee engagement through mediating variable, job satisfaction.

**Figure 2:**
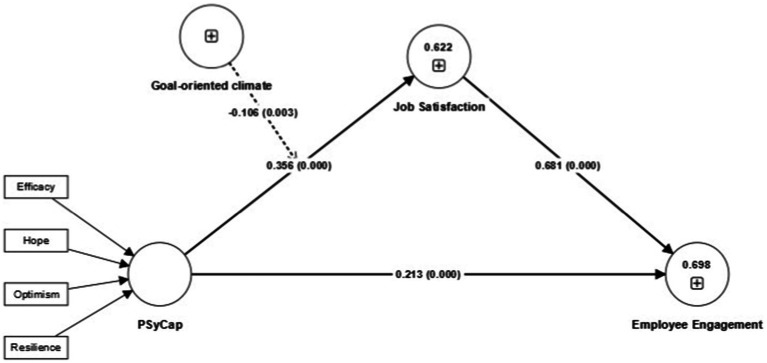
Structural model.

As Figure 2 shows, the *R*^2^ values of the endogenous constructs are above the 0.10 threshold (Falk and Miller, 1992). Specifically, in our study an *R*^2^ = 0.698 (substantial value) and *R*^2^ = 0.622 (moderate value) was obtained, which implies that PsyCap and Goal-oriented Climate, through their effect on Job Satisfaction, explain the 69.8% of employee engagement, and 62.2% of job satisfaction is explained by the PsyCap and the Goal-Oriented Climate.

Finally, according to Cohen (1998), the size of the effect of Job Satisfaction on Employee Engagement (*f*^2^ = 0.891) and Goal-oriented Climate effect on Job Satisfaction (*f*^2^ = 0.497) are high (*f*^2^ ≥ 0.35), but the size of the PsyCap effects on Job Satisfaction (*f*^2^ = 0.228) is moderate (0.15 ≤ *f*^2^ < 0.35), while the rest are weak.

## 4 Discussion

The objective of this study is to investigate how the multiplicative effect of resources (personal resources and a goal-oriented climate) among public employees influences their satisfaction and engagement at work. To achieve this objective, an analytical model was employed, examining how psychological capital’s impact on engagement is channeled through job satisfaction, while also being influenced by the climate of goal orientation.

The first hypothesis suggests that there is a positive effect of PsyCap on employee engagement. The results have shown a direct positive influence of PsyCap on engagement. This evidence supports the notion that higher levels of psychological capital among public employees are associated with higher levels of engagement in their work. These findings align with those established previously by Rothmann (2008) and Simons and Buitendach (2013), and underscore the importance of personal resources in the motivational process of the JD-R theory (Bakker and Demerouti, 2017).

The second hypothesis suggested that job satisfaction would positively influence engagement. The results have demonstrated a direct positive influence of job satisfaction on engagement (0.681***). These findings underscore the significance of job satisfaction as a contributor to work engagement. They support the significant role of job satisfaction in work engagement (Yalabik et al., 2017; Zhang and Li, 2020).

The third hypothesis suggests that PsyCap will have a positive influence on job satisfaction. The results of this study indicate that public employees with higher levels of PsyCap are the ones who have shown the highest job satisfaction. This finding supports the proposition by Kong et al. (2018) regarding the influence of employees’ personal resources on their own satisfaction and emotional response.

The fourth hypothesis posits that job satisfaction plays a mediating role between PsyCap and employee engagement. The data clearly reflect this mediating effect of job satisfaction. These findings align with the propositions of authors such as Azanza et al. (2013), Badran and Youssef-Morgan (2015), and Wefald and Downey (2009), who advocate for the potential mediating effect of job satisfaction.

The fifth hypothesis asserts that the Goal-oriented Climate moderates the relationship between PsyCap and Job Satisfaction, indicating that the relationship between PsyCap and Job Satisfaction is weaker in a goal-oriented climate. The data support this hypothesis but add the nuance that the moderating effect of the goal-oriented climate is negative. This suggests that the presence of a goal-oriented climate in public administration will negatively influence the job satisfaction of employees with high personal resources. These results support the importance of the goal orientation theory (Vandewalle et al., 2001), which emphasizes the relevance of this perception among public employees, albeit with the caveat that this relevance should be minimized as it has a negative impact.

These findings corroborate the importance of job resources within the motivational process of the JD-R theory (Bakker and Demerouti, 2017), further emphasizing the added value of having been conducted with a sample of public sector employees characterized by high geographical homogeneity and great stability in their contracts. In this way, they support the results of previous seminal research lines on the effects of resources on motivational response and employees’ organizational attachment, highlighting that these findings occur not only in private administration entities but also in different work contexts. It is noteworthy that the aggregation of resources does not necessarily yield a positive outcome for the organization, regardless of the population characteristics where this occurs. Consequently, the outcomes of this study provide a basis for delineating the following theoretical and practical implications.

### 4.1 Theoretical implications

The theoretical implications derived from these findings are substantial. Firstly, they corroborate prior research findings that advocate for the significance of both personal and job resources in the motivational process, while extending this conclusion to the specific context of the Public Administration sector. Numerous authors such as Cooke et al. (2018), Bailey et al. (2015), Vigoda-Gadot et al. (2013), and Zahari and Kaliannan (2022) have previously underscored the relevance of resources in fostering employee engagement. However, investigating this relationship within public administrations remained a challenge (Saks, 2006; Costantini et al., 2017). The present study addresses this challenge and identifies a direct positive relationship between resources and the satisfaction and engagement of public employees.

The second theoretical implication arises from the proposed model itself. Previous studies examining the influence of resources on engagement have consistently posited a direct relationship between them. However, the model analyzed in this study suggests that the influence of personal resources intersects with various other factors, such as the goal-oriented climate and individual emotional state (job satisfaction), to shape an employee’s engagement with their organization. These findings align with prior research suggesting the mediating role of job satisfaction (Wefald and Downey, 2009; Azanza et al., 2013; Badran and Youssef-Morgan, 2015), but offer a more comprehensive perspective on the motivational process proposed by JD-R theory (Bakker and Demerouti, 2017; Bakker et al., 2023). Based on the results of this study, the influence of resources on engagement will largely depend on the effect they have on each employee’s individual assessment of job satisfaction.

The final theoretical implication pertains to the cumulative effect of accumulating resources on the motivational process. The JD-R model itself posits that resources and demands have a multiplicative impact on employee well-being (Bakker et al., 2023). Most theories proposed for analyzing the work context, which have examined the influence of resources on engagement or job satisfaction in the motivational process, have suggested positive effects on these variables (Vandewalle et al., 2001; Bakker and Demerouti, 2017; Ryan and Deci, 2017).

This may lead us to infer that the summation of these resource effects will also yield positive outcomes in employees’ emotional responses (Bakker et al., 2023). However, the negative modulation effect of goal orientation on the influence of PsyCap on public employees’ job satisfaction indicates that, despite the direct correlation between psychological capital and goal orientation having a positive effect, the interaction of both resources has a negative impact on public employees’ job satisfaction.

### 4.2 Practical implications

The practical implications of the findings are varied and hold importance for organizational management and human resources practices in public administrations.

The first implication highlights the need to foster job satisfaction among public sector employees to generate high levels of engagement with their organization. Despite being a group with very stable and beneficial working conditions, the findings of this study suggest the need to increase their satisfaction through people management to affect their engagement. Thus, a significant portion of the effect on engagement in this group depends on both personal resources (PsyCap) and job-related resources (goal orientation climate). This confirms the importance of resources impacting job satisfaction even among public employees; otherwise, their influence on fostering engagement could be very limited. Therefore, the development of intervention plans aimed at improving resources among public employees should prioritize increasing job satisfaction over the pursuit of efficiency or effectiveness.

The second practical implication of this study concerns the negative multiplicative effect of psychological capital and goal orientation climate. In the effort to foster engagement among public sector employees by improving their job satisfaction, it is imperative to recognize that increasing all job resources does not necessarily produce positive results. This finding suggests that both individual characteristics of employees and contextual characteristics specific to public employees in a particular area must be considered. Thus, the combination of high levels of PsyCap with high goal orientation among employees can have a detrimental effect on their job satisfaction and, consequently, on their engagement. Therefore, these results underscore the importance of organizations understanding the idiosyncrasies of their human capital and conducting a thorough assessment of their resources and interests before designing any resource improvement programs. Failure to do so may result in interventions aimed at increasing specific job resources having initially unintended adverse effects.

### 4.3 Limitations and future research directions

As for limitations of the present study, four main ones can be identified. Firstly, there is a limitation associated with the method used in this study, as the information was collected through online questionnaires (self-reports), which could potentially be affected by common method variance (Simmering et al., 2015; McGonagle, 2017).

Secondly, there is a limitation that needs to be considered when generalizing the findings. These results were obtained from a sample of subjects from a very specific population of public employees in Spain. This population possesses cultural and social characteristics that require caution when generalizing the results to other contexts.

Thirdly, the present study employs a cross-sectional design. This makes it difficult to analyze the effect of temporal causality between variables.

Lastly, all analyses have been conducted at the individual level, considering the scores of each subject. It would be advisable to sample in a way that allows for the analysis of these results from group or organizational perspectives as well (Bakker et al., 2023).

Considering the limitations, four future lines of inquiry can be proposed to deepen the relationship between these variables. Firstly, it would be advisable to compare the results obtained from self-report instruments with data collected through other qualitative methodologies such as direct observation, employee interviews, or the compilation of objective data related to well-being, such as employee turnover rates or individual productivity.

Another prospective avenue of investigation suggests replicating this model in different cultural contexts. This would allow for contrasting the results of the present study with groups of public employees exhibiting different characteristics or even with samples from private enterprises (Costantini et al., 2017; Mañas et al., 2018). In addition, it would be appropriate to also use other analysis methods such as covariance-based SEM (CB-SEM), to verify the consistency of the results, as well as analyze the presence of other moderating variables that could influence the relationship between PsyCap and job satisfaction, such as organizational support, organizational culture and leadership.

A third future line of research should consider longitudinal research designs. Such designs would enable the temporal evolution of the relationships between the variables proposed in this study to be described.

Lastly, conducting sample collections that permit the comparison of shared perceptions among team employees would be beneficial. Designing studies that analyze the proposed variables at different levels (individual, group, and even organizational) would facilitate the understanding of the relationship between the study variables. This approach would also aid in result generalization and provide greater insight into the mechanisms of the motivational process within organizations.

## 5 Conclusion

In conclusion, this study offers a novel perspective on the factors that influence job satisfaction and employee engagement in the public sector. Grounded in the personal resources of employees (PsyCap), the impact of these resources on the level of engagement is contingent, in part, upon their ability to foster job satisfaction among public employees. Within this mediating process, the configuration of the organizational climate plays a significant role. The findings have demonstrated that the presence of a goal-oriented climate in this context negatively influences job satisfaction among public employees with higher levels of PsyCap, subsequently affecting their level of engagement with the organization.

## Data availability statement

The datasets presented in this study can be found in online repositories. The names of the repository/repositories and accession number(s) can be found at: http://hdl.handle.net/10835/14550.

## Ethics statement

The studies involving humans were approved by University of Almeria Bioethics committee. The studies were conducted in accordance with the local legislation and institutional requirements. The participants provided their written informed consent to participate in this study. Written informed consent was obtained from the individual(s) for the publication of any potentially identifiable images or data included in this article.

## Author contributions

PD-F: Conceptualization, Formal analysis, Investigation, Resources, Validation, Visualization, Writing – original draft, Writing – review & editing. GC: Data curation, Formal analysis, Investigation, Methodology, Validation, Writing – original draft. BH-S: Funding acquisition, Investigation, Resources, Writing – review & editing. JS-G: Conceptualization, Funding acquisition, Investigation, Project administration, Resources, Writing – review & editing. MM-R: Conceptualization, Investigation, Project administration, Supervision, Validation, Writing – original draft, Writing – review & editing.
